# Supplementary material for: Novel Benzothiazole Boc-Phe-Phe-Bz Derivative Dipeptide Forming Fluorescent and Nonlinear Optical Self-Assembled Structures
Source: Molecules. 2025 Feb 18;30(4):942. doi: 10.3390/molecules30040942 (PMC11858552; doi:10.3390/molecules30040942)
Supplement: Supplementary file 1 [file molecules-30-00942-s001.zip › molecules-3454911-supplementary.pdf]

Supplementary material

Novel Benzothiazole Boc-Phe-Phe-Bz Derivative Dipeptide Forming Fluorescent and Nonlinear Optical Self-Assembled Structures

Rosa M. F. Baptista [1,*], Daniela Santos [1], N. F. Cunha [1], Maria Cidália R. Castro [2], Pedro V. Rodrigues [2], Ana V. Machado [2], Michael S. Belsley [1] and Etelvina de Matos Gomes [1,*]

S1. ^{1}H NMR and ^{13}C NMR spectra

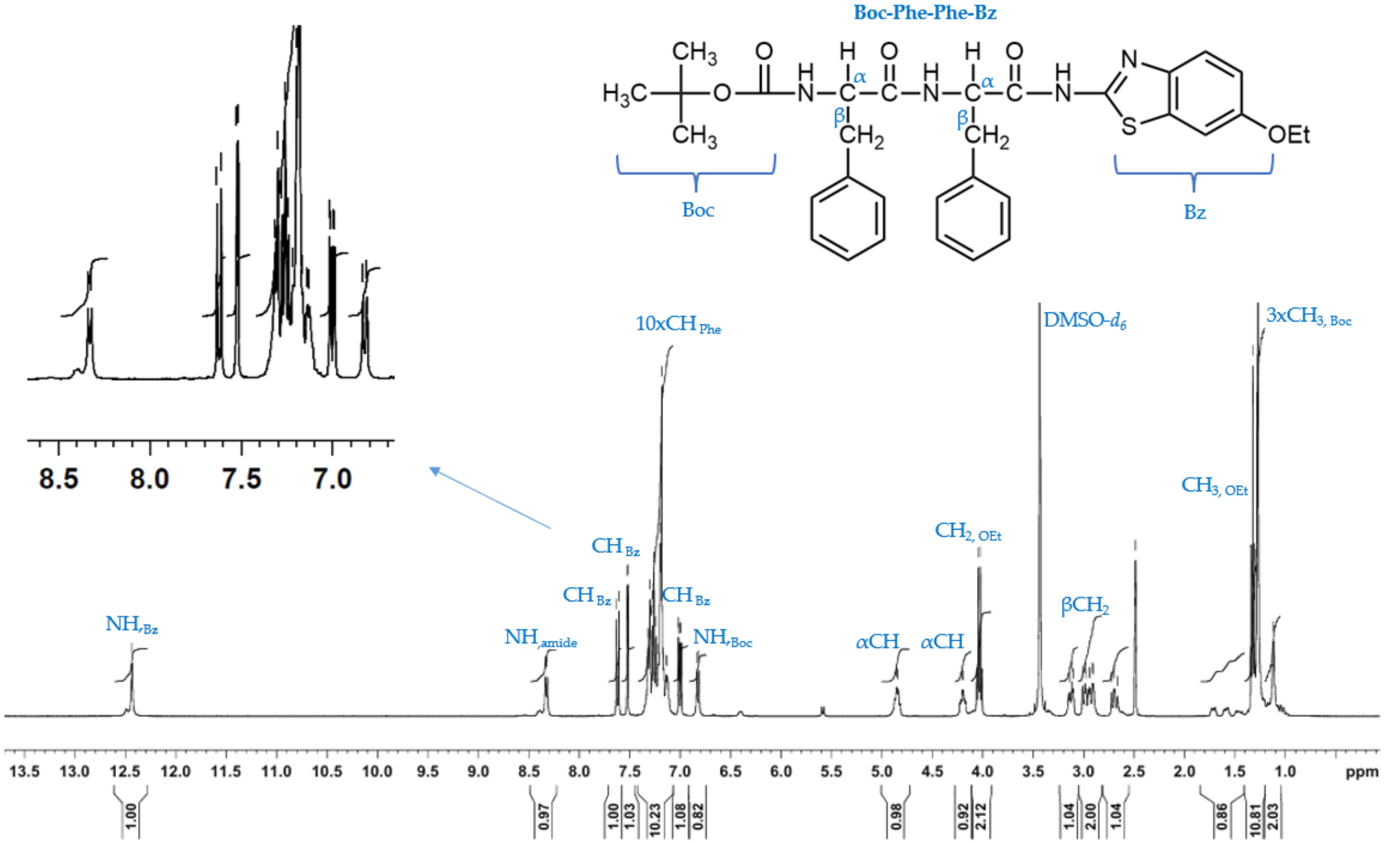

Figure S1. ^{1}H NMR spectrum of the Boc-Phe-Phe-Bz dipeptide in DMSO-d_6 at room temperature, measured at 400 MHz.

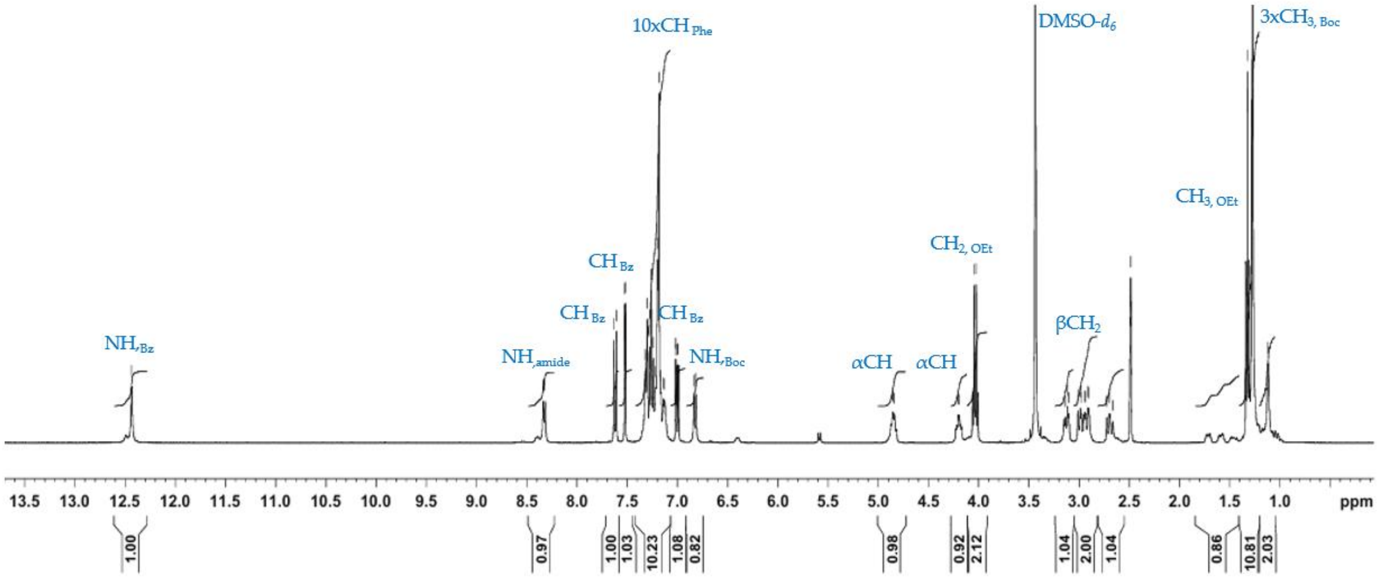

Figure S2. ^{13}C NMR spectrum of the Boc-Phe-Phe-Bz dipeptide in DMSO-d_6 at room temperature, measured at 100.6 MHz.

S2. Fourier-transform infrared spectroscopy spectra

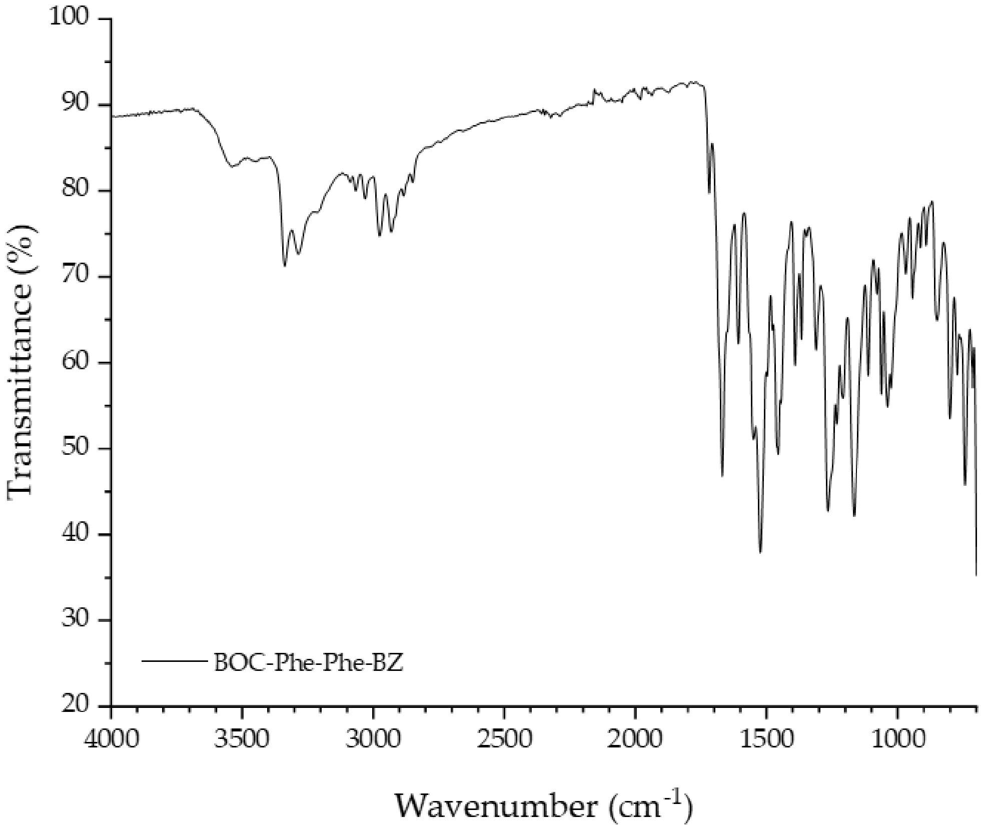

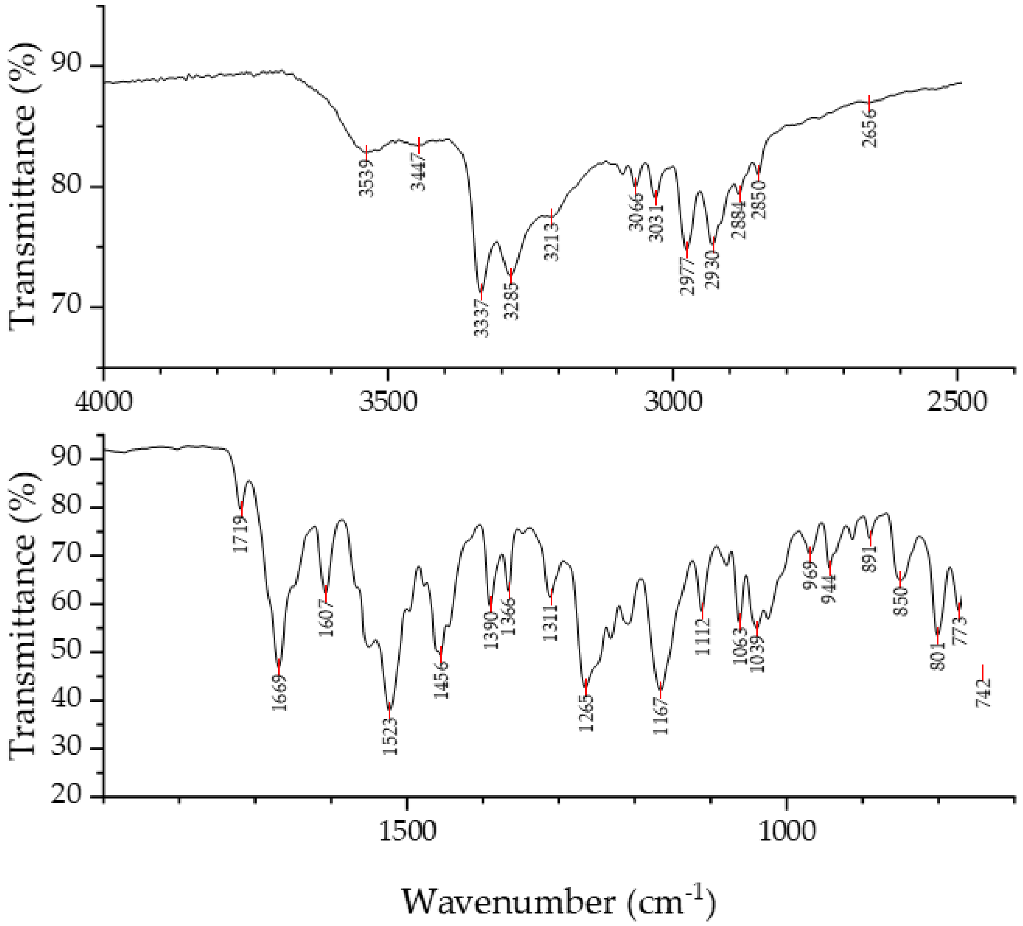

Figure S3. FTIR spectra of the Boc-Phe-Phe-Bz dipeptide measure in a PerkinElmer Spectrum 100 spectrometer in ATR mode with 16 accumulations, 4 cm^{-1} resolution and a range of 4000-700 cm^{-1}.

Fourier transform infrared spectroscopy (FTIR) analysis was performed in a Jasco FT/IR-4X (JASCO Corp., Tokyo, Japan) in ATR mode with 32 accumulations, 4 $cm^{−1}$ resolution, and a range of 4000–700 cm^{-1}. The region 3500–3200 cm^{-1} is important for N-H vibrations, where the band at 3285 cm^{-1} indicates the presence of strongly hydrogen-bonded NH groups, whereas the band at 3337 cm^{-1} indicates that not all NH groups are involved in intermolecular hydrogen bonding. The bands between 2930-3031 cm^{-1} are associated to the aromatic C-H vibrations and the band at 1535 cm^{-1} corresponds to the C=C bending bands of the aromatic ring of the phenylalanine. The Boc-Phe-Phe-Bz dipeptide presents two types of amide groups (C=O amide) at 1719, 1669, and 1607 cm^{-1} suggesting that the peptide adopts a broadly hydrogen-bonded network in the nanobelts. Concerning the benzothiazole ring, the aliphatic C-H stretching: 2850-2950 $cm^{−1}$ (vibrations of the C-H bonds in the ethoxy group) the C=N stretching characteristic of the heterocyclic group at 1607 $cm^{−1}$, C-O-C stretching at 1050-1150 $cm^{−1}$ associated to the asymmetric vibration of the ethoxy group, and C-S stretching at 742-773 cm^{-1} [1,2].

S3. Differential scanning calorimetry (DSC) thermal gravimetric analysis (TGA)

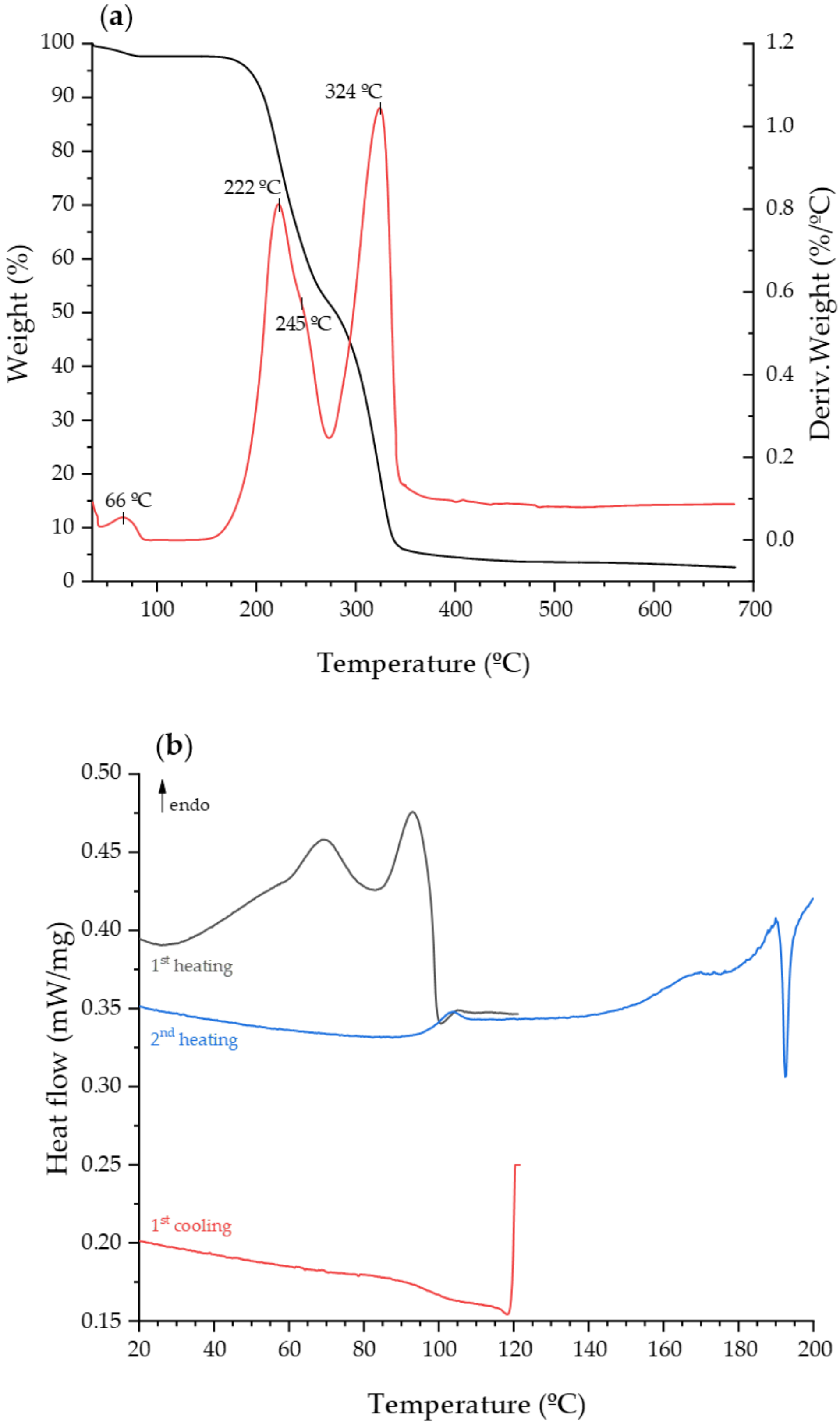

Figure S4. TGA (a) and DSC (b) thermograms of the Boc-Phe-Phe-Bz dipeptide.

Figure SI4 depicts the TGA and DSC thermograms of Boc-Phe-Phe-Bz. The TGA spectrum shows an initial mass loss of 2.3% up to 90 ºC that corresponds to the water molecules loss. Above 140 ºC there is a significant mass loss which corresponds to the degradation of the Boc group from the crystalline compound. The degradation mechanism occurs in three stages, as visible by the presence of three derivative weight peaks: at 222, 245 and 324 ºC, with a degradation weight of 21, 37 and 80 %, respectively. On DSC analysis, the first

heating shows the release of volatile compounds (such as water molecules) up to 100ºC, which is not detected on the second heating, and the degradation of the compound initiates at 150ºC. These results are in agreement with TGA.

References

1. Baptista, R.M.F.; de Matos Gomes, E.; Raposo, M.M.M.; Costa, S.P.G.; Lopes, P.E.; Almeida, B.; Belsley, M.S. Self-assembly of dipeptide Boc-diphenylalanine nanotubes inside electrospun polymeric fibers with strong piezoelectric response. *Nanoscale Advances* **2019**, *1*, 4339-4346, doi:10.1039/C9NA00464E.
2. Bera, S.; Jana, P.; Maity, S.K.; Haldar, D. Inhibition of fibril formation by tyrosine modification of diphenylalanine: crystallographic insights. *Cryst Growth Des* **2014**, *14*, 1032-1038.
